# Remembering the past – studies on evolution done by the genetics group at Universidade Federal do Rio Grande do Sul (UFRGS)

**DOI:** 10.1590/1678-4685-GMB-2017-0049

**Published:** 2018-03-19

**Authors:** Francisco M. Salzano

**Affiliations:** 1 Departamento de Genética, Instituto de Biociências, Universidade Federal do Rio Grande do Sul, Porto Alegre, RS, Brazil

**Keywords:** History, genetics research, evolutionary genetics, Porto Alegre, Brazil

## Abstract

After a brief introduction about the factors that are involved in science development, and world and Brazilian evolutionary genetics, the studies developed in Porto Alegre in this area were reviewed. Four periods in the development of this group were distinguished: (a) Origins and first expansion (1949-1961); (b) Second expansion (1962-1988); (c) Third expansion (1989-2001); and (d) The last 15 years (2002-present). The international Porto Alegre Biological Evolution Workshops (PABEWs), with five biannual events from 2007 o 2015, were also mentioned. The final message stressed the importance of the maintenance of this and other Brazilian groups of research through adequate finance and recognition.

## Factors involved in science development

What conditions the formation and maintenance of a research group? We could conveniently classify them in two components. The first is environmental. Important scientific investigation cannot thrive in a hostile environment, characterized by violence (wars, within-nations arrest due to power contests), poor economic development, or general ignorance about the importance of science. The second is mainly historical; it depends on the presence of the right person, in the right place. All societies have persons endowed with high intelligence and adequate drive to pursue a successful scientific career. However, they may never have the opportunity of adopting it due to the absence of adequate conditions. Another crucial factor is continuity. Initially favorable situations may be of short duration, and the entire enterprise may then fail to be maintained. In addition, the formation of a scientist is a slow process. It depends on an initial adequate interpersonal relationship in the educational process, which is of an artisan type. The assimilation of the abstract concepts involved requires time and may be subjected to distracting interests. Therefore, research groups lasting for many years are rarely numerous.

It is in this context that we should view the history of our group that, despite fluctuations in the economic conditions and governmental policies, maintained a pattern of excellence not common in Third World countries. This continuity can be documented through the years that elapsed since Doctoral titles were awarded to the main contributors of our research group (Table 1). The dates extend from 1954 to 2009, more than half a century, with a steady rhythm of growth during the 1970s to 1990s.

**Table 1:** List of the main researchers involved in evolutionary studies in Porto Alegre with the dates they obtained their Ph.D. degrees^1^.

	Researcher	Main organisms studied	Date of Ph.D. award
1.	Antonio R. Cordeiro	Animal	1954
2.	Francisco M. Salzano	*Homo sapiens*	1955
3.	Edmundo K. Marques	Animal	1968
4.	Fernando J. da Rocha	*Homo sapiens*	1970
5.	Maria Irene B. Moraes Fernandes	Plant	1971
6.	Casemiro V. Tondo	*Homo sapiens*	1971
7.	Helga Winge	Plant	1971
8.	Maria José Melo e Freitas	*Homo sapiens*	1971
9.	Aldo M. Araújo	Animal	1973
10.	Margarete S. Mattevi	Animal	1974
11.	Marly Napp	Animal	1975
12.	Alice K. Oliveira	Animal	1979
13.	Tania A. Weimer	*Homo sapiens*	1980
14.	Maria Helena L. P. Franco	*Homo sapiens*	1980
15.	Mara H. Hutz	*Homo sapiens*	1981
16.	Vera L. S. Valente	Animal	1984
17.	Suzana C. Molina	Plant	1984
18.	Sidia M. Callegari-Jacques	*Homo sapiens*	1985
19.	Thales R. O. Freitas	Animal	1990
20.	Márcia Margis-Pinheiro	Plant	1993
21.	Rogério Margis	Plant	1993
22.	Maria Cátira Bortolini	*Homo sapiens*	1996
23.	Tatiana T. Souza-Chies	Plant	1996
24.	Sandro L. Bonatto	*Homo sapiens*	1997
25.	Elgion L. S. Loreto	Animal	1997
26.	Karen L. Haag	Animal	1997
27.	Loreta B. Freitas	Plant	1997
28.	Eliane K. Santos	Plant	1999
29.	Fernanda Bered	Plant	1999
30.	Eduardo Eizirik	Animal	2002
31.	Nelson J. R. Fagundes	*Homo sapiens*	2007
32.	Andréia C. T. Zolet	Plant	2009

1 Flavio Lewgoy never obtained a formal Ph.D. degree.

## World and Brazilian evolutionary genetics

The beginning of evolutionary genetics can be conveniently dated to 1908, when the fundamental principle developed by G. H. Hardy (1877-1947) and W. Weinberg (1862-1937), established what would be the fate of genes in populations. What could be labeled as the period of classical genetics extends from 1900 to 1953 (Carlson, 2004), and the formation of the UFRGS genetics group occurred during the fusion of Darwinism and Mendelism, in the fertile two decades (1930-1950) of the development of the synthetic theory of evolution (Herrera *et al.*, 2016).

The subsequent years were characterized by a phenomenal development in the area of genetics, starting in 1953 with the elegant DNA molecule model devised by James D. Watson (born in 1928) and Francis H. C. Crick (1916-2004), to which Rosalind E. Franklin (1921-1958) and Maurice H. F. Wilkins (1916-2004) significantly contributed.

The progress in the area of evolutionary genetics was in great part due to the development of laboratory and analytic methods for the study of population variability, that evolved from immunological to biochemical, to molecular determinations, that then were extensively analyzed by computer and bioinformatics programs.

What was happening in Brazil during these years? Well, no Brazilian received the Nobel Prize for his/her studies in evolutionary genetics. However, Brazilian researchers followed closely these tendencies, furnishing valuable data of worldwide importance. I have reviewed this information along the years (Salzano, 1979, 2011, 2012) and the reader is referred to these publications for more details.

## Studies in Porto Alegre

### Origins and first expansion (1949-1961)

It all started with a young man of 24 years of age, Antonio Rodrigues Cordeiro, student of the Natural History Course, School of Philosophy, University of Rio Grande do Sul (at the time it was not yet a Federal University). After a practical class on *Drosophila melanogaster* he decided to verify whether these flies occurred also in our environment. He then wrote to Crodowaldo Pavan, at the time an Assistant Professor at the University of São Paulo, who sent him a detailed letter on the way that these flies could be collected and raised in the laboratory.

One year later, Theodosius Dobzhansky, one of the scholars responsible for the development of the synthetic theory of evolution, arrived in São Paulo for a stay of one year, and Cordeiro, together with A. G. L. Cavalcanti (who worked in Rio de Janeiro) were selected for a one-year fellowship to form (with others) the team responsible for the research to be coordinated by Dobzhansky.

In September, 1949, A. R. Cordeiro returned from São Paulo. He was already an Assistant Professor at UFRGS, and with the decisive support of the then Director of the School of Philosophy, Bernardo Geisel (1901-1985), he organized a small laboratory in the basement of the Law School. The first expansion of genetics research in Porto Alegre occurred in 1953, when we moved to a new building with much more space, which was the seat of a newly created Institute, the Institute of Natural Sciences. During the period that elapsed until the new move, in 1961, significant research was performed, which is summarized in Cordeiro and Salzano (1961).

My relationship with the group started as a voluntary student, before my graduation, in 1950. In the next year I received a fellowship from the University of São Paulo, and during one I year worked there under the guidance of Antonio Brito da Cunha, Crodowaldo Pavan, and Hampton L. Carson, from the Washington University, Missouri, USA. After returning to Porto Alegre in 1952 I was invited to work as an Instructor at UFRGS, the institution where I remained until now. Four years later (1956) I obtained a one-year post-doctoral fellowship to work at the University of Michigan, Ann Arbor, under the supervision of James V. Neel, and after my return started to work on human population genetics, that continues up to the present.

Selected aspects of the research in evolutionary genetics performed at the time will now be briefly summarized. Key persons involved besides Cordeiro and myself were, in alphabetical order of the first name, Casemiro V. Tondo, Edmundo K. Marques, Flavio Lewgoy, Helga Winge, and Marly Napp.

Recessive alleles concealed due to dominance in *Drosophila* could be detected due to a skillful method of crossings. These were performed by the group using *Drosophila willistoni,* and the main results were reported in Burla *et al.* (1949), Pavan *et al.* (1951), Cordeiro (1952), and Cordeiro and Dobzhansky (1954).

In 1956 the laboratories of biophysics and genetics merged, and Casemiro V. Tondo became a full member of the group. Cordeiro was aware of the need to develop new methods of genetic analysis, and together with Tondo and Flavio Lewgoy, a biochemist, produced a series of four articles all entitled Biophysical Genetics (Tondo and Cordeiro, 1956; Lewgoy and Cordeiro, 1958; Tondo *et al.*, 1959; Cordeiro *et al.*, 1960a). They applied chromatography, paper electrophoresis, and spectrophotometric methods to characterize homo and heterozygous strains, as well as interracial hybrids of *Drosophila willistoni*.

Concomitantly, inversion polymorphisms and the peculiarities of the bocainensis cryptic group of species were investigated by Da Cunha *et al.* (1953) and Salzano (1956). The fate of chromosome inversions experimentally released in populations where they had been previously absent was examined by Cordeiro *et al.* (1960b).

In another area, the first studies in plants were performed by Winge (1959), on the cytotaxonomy and polymorphism of the genus *Alophia* (Iridaceae).

Studies in human evolutionary genetics had started in 1958, and a series of papers reporting the investigations performed among the Kaingang Amerindians of Rio Grande do Sul were published ([Bibr B43] [Bibr B44] [Bibr B45] Salzano, 1961a-d).

### Second expansion (1962-1988)

The transfer of the Department’s seat from the UFRGS Central Campus to a new and much expanded space, located in three floors of a commercial building situated in the center of the city, opened considerable new opportunities for expansion, both in terms of personnel and research.

Considering first studies in animals, the investigations with *Drosophila* continued with vigor; examples are as follows: 1. The finding of hybrids between *D. willistoni* and *D. paulistorum* (Winge and Cordeiro, 1963), first denied by some researchers, but afterwards firmly confirmed; 2. the question of the adaptation of *D. willistoni* to an environment with high background radiation (Cordeiro *et al.*, 1973); 3. biochemical variability (esterases, alcohol dehydrogenases) in natural populations (Napp and Cordeiro, 1978; Albuquerque and Napp, 1981; Oliveira and Cordeiro, 1985; Uriarte and Napp, 1988); 4. ecology in *D. incompta* (Hofmann and Napp, 1984); and 5. chromosomal polymorphism in *D. willistoni* (Valente and Araújo, 1986). Studies on the butterfly genus *Heliconius* were performed by Lima and Araújo (1982), and Menna-Barreto and Araújo (1985). Going from insects to mammals, investigations were done on *Scapteromys* (Freitas *et al.*, 1984), *Nectomys* (Maia *et al.*, 1984), and *Deltamys* (Sbalqueiro *et al.*, 1984). Also, a long-term relationship between the Genetics Departments of the Federal Universities of Rio Grande do Sul and Pará resulted in four articles about the genetic variability of Amazonian buffaloes and non-human primates (*Cebus*, *Alouatta*). Details can be obtained with Horacio and Maria Paula Cruz Schneider, in Belém.

Plant evolutionary genetics involved: 1. Chromosome relationships in the genera *Paspalum* and *Axonopus* (Gramineae), (Moraes-Fernandes *et al.*, 1968, 1973, 1974; Hickenbick *et al.*, 1975); 2. Altitude and cyanogenesis in the white clover *Trifolium repens* (Araújo, 1976); and 3. Biochemical variability in the rubiacea *Relbunium* (Porto *et al.*, 1977; Cavalli-Molina and Winge, 1988).

Extensive work during this period was done on human populations, and the corresponding list of publications is too extensive to be given in full. Key persons at this time were (again by alphabetical listing of the first name) Casemiro V. Tondo, Fernando J. da Rocha, Mara H. Hutz, Maria Helena L. P. Franco, Maria José de Melo e Freitas, Sidia M. Callegari-Jacques, and Tania A. Weimer. I published studies resulting from field work on Amerindians, including the Kaingang, Xavante, Kayapo, Krahó, Macushi, Wapishana, Yanomama, Ticuna, Pacaás Novos, Sateré-Mawé, and Içana River populations. These studies, together with others done by our group and by additional researchers, were considered in a global way by Salzano and Callegari-Jacques (1988). Non-Amerindian populations were also investigated, including communities from Porto Alegre, Natal, Aracajú, and several Amazonian locations. Special mention should be made of the discovery of Hemoglobin Porto Alegre (Tondo *et al.*, 1963) due to its peculiarity (*in vitro* polymerization into octamers and dodecamers), as well as by its intermediate frequency, not being very rare nor much frequent. These results, together with those presented in the context of the next historical period, were reviewed in Salzano and Bortolini (2002).

### Third expansion (1989-2001)

With the increase in the number of persons and studies that occurred in this and the following period, references would have to be even more selective than those of the previous sections. Starting with animal evolutionary genetics, 12 articles reporting *Drosophila* results have been published; including *D. nebulosa*, *D. maculifrons*, *D. willistoni*, *D. paulistorum*, *D. simulans*, *D. polymorpha*, *D. tripunctata*, and many other species. A general review about the transposable elements in Neotropical *Drosophila* was published by Loreto *et al.* (1998). Work on other insects were much less numerous, including aphids, *Chauliognathus* (Coleoptera), and *Dryas iulia* (Lepidoptera; Haag *et al.*, 1993). Studies in rodents were numerous, involving work on *Deltamys*, *Ctenomys*, *Oryzomys*, *Oligoryzomys*, *Akodon*, *Rhipidomys*, *Delomys*, *Nectomys*, and *Decomys* genera, as well as two species of bats and Neotropical cats (Eizirik *et al.*, 1998). The association between the Federal Universities of Pará and Rio Grande do Sul (see the previous section) resulted in nine articles published between 1989 and 1995 on biochemical protein polymorphisms, and seven on chromosome markers, all in New World primates. Details about this latter data set can be obtained from Julio Cesar Pieczarka and/or Cleusa Y. Nagamachi in Belém.

In plants, additional studies in *Relbunium*, *Ilex*, and *Leucaena* (Leguminosae; Cardoso *et al.*, 2000) were published.

As far as humans are concerned, as mentioned previously, the results obtained on non-Amerindian populations were extensively reviewed in Salzano and Bortolini (2002). Special mention can be made of a series of articles that resulted from a joint program of our group with the Biological Anthropology Department, School of Humanities and Education Sciences, Universidad de la República, Montevideo, as for the study by Sans *et al.* (1997). This period was especially fruitful with regard to the genetic investigations on Amerindian populations, performed in association with a network of colleagues from Latin America, North America, Europe and Asia. No less than 47 different groups (Table 2) were investigated on various aspects related to protein and DNA markers. Details can be provided on request. Two especially important, widely cited papers were those of Bonatto and Salzano (1997a, b) who, through a sophisticated analysis of Amerindian mitochondrial DNA sequences, arrived at the conclusion that the prehistoric peopling of the Americas occurred due to a single and early migration.

**Table 2:** List of the Amerindian populations for which genetic data have been reported by members of the Porto Alegre group in collaboration with many colleagues from Latin America, North America, Europe, and Asia, in the period between 1989 and 2001.

Amerindian populations			
1.	Ache	25.	Mapuche
2.	Apalaí-Wayana	26.	Mataco (Wichi)
3.	Arara	27.	Mundurucu
4.	Araweté	28.	Mura
5.	Asurini-Koatinemo	29.	Mvskoke
6.	Asurini-Trocará	30.	Pacaás Novos
7.	Ayoreo	31.	Palikour
8.	Carrier-Sekani	32.	Parakanã
9.	Choroti	33.	Pilagá
10.	Cinta Larga	34.	Pukany
11.	Galibi	35.	Sateré-Mawé
12.	Gavião	36.	Suruí
13.	Guarani	37.	Tehuelche
14.	Içana River	38.	Tenharim
15.	Jamamadi	39.	Ticuna
16.	Kaingang	40.	Tiriyó
17.	Kararahô	41.	Toba
18.	Karitiana	42.	Urubu-Kaapor
19.	Kayapo	43.	Wai Wai
20.	Krahó	44.	Waiãpi
21.	Kubenkokre	45.	Wayana
22.	Lengua	46.	Xavante
23.	Macushi	47.	Zoró
24.	Makiritare		

### The last 15 years (2002-present)

The amount of publications presented in this period is very large, and a comprehensive list is impossible within the limits of this review. Therefore, only general information is provided in [Table t3] [Table t4] [Table t5] Tables 3-6, with indications of the years in which they were published and the colleagues who could give more details about them.

**Table 3 t3:** Selected examples of evolutionary studies of a general nature and on microorganisms performed by members of the Porto Alegre group (2002-present).

Year	Nature of the study	Contact
2007	Phylogenomics of mycoplasmas	S.L. Bonatto
2007, 2010	Phylogeny, Ciliophora, Peritrichia	E. Eizirik
2008	Molecular markers, populations, and geography	S.L. Bonatto, E. Eizirik, T.R.O. Freitas
2011	Rates of evolution, porcine parvovirus	S.L. Bonatto
2013/14	Population expansion and genome reduction in microsporidia	K.L. Haag
2015	Retrovirus, felids, identification and characterization	E. Eizirik
2016	Hepatitis B virus distribution in an ecological context	N.J.R. Fagundes, F.M. Salzano
	Refuge theory, geographical barriers, and biome variability in the Atlantic Forest	L.B. Freitas, S.L. Bonatto

**Table 4 t4:** Selected examples of plant evolutionary studies performed by members of the Porto Alegre group (2002-present).

Year	Nature of the study and organisms	Contact
2003-present	Extensive number of different types of proteins, their taxonomic distribution, phylogeny and phylogeography	M. Margis-Pinheiro, R. Margis, L.B. Freitas, A.C. Turchetto-Zolet
2009	Aquifoliaceae,*Ilex* phylogeny	T.T. Souza-Chies
2016	Asteraceae,*Gerbera*, phylogeny and biogeography	T.T. Souza-Chies
2007-2016	Bromeliaceae,*Vriesea*,*Bromelia*,*Aechmea* molecular variability	F. Bered
2011, 2015	Iridaceae,*Sisyrinchium*, cytogenetic distribution, phylogeny	E.K. Santos, T.T. Souza-Chies
2012	Lamiaceae,*Cunila*, phylogeny	T.T. Souza-Chies
2016	Myrtaceae, phylogeography	R. Margis
2005-present	Passifloraceae, extensive studies on large number of species, and aspects of their evolution, phylogeny, and phylogeography	L.B. Freitas
2006, 2008, 2015, 2016	Poaceae,*Briza*,*Paspalum*,*Eryochrysis*,*Saccharum*, phylogeny, hybridization	T.T. Souza-Chies
2006-present	Solanaceae,*Petunia*, large number of studies on their phylogeny and phylogeography	L.B. Freitas

**Table 5 t5:** Selected examples of animal evolutionary studies performed by members of the Porto Alegre group (2002-present).

Year	Nature of the study and organism	Contact
2006, 2013, 2015	General, Paired box (PAX) gene family, origins, evolvability, phylogeny	M.C. Bortolini, F.M. Salzano
2008, 2009, 2016	Plathyhelminthes, Cestoda,*Echinococcus*, variability, phylogeography	K.L. Haag
2010	Mollusca,*Phyllocaulis*, phylogeny	S.L. Bonatto
2004	Arthropoda, Coleoptera,*Chauliognathus*, phylogeny	A.M. Araújo
2011	Arthropoda, Lepidoptera,*Heliconius*, kin recognition, evolutionary implications	A.M. Araújo
2002-2016	Arthropoda, Diptera, Drosophilidae,*Drosophila*, Culicidae,*Anopheles*, extensive investigation on all aspects of evolutionary diversification in many species, phylogeny, transposon identification and coevolutionary relationship	V.L.S. Valente, E.L.S. Loreto
2011	Vertebrates, Siluriformes, Neoplecostominae, Hypoptopomatinae, phylogeny	S.L. Bonatto
2006, 2012	Reptilia,*Bothrops*,*Corallus*, Dipsadidae, phylogeny	S.L. Bonatto
2009, 2016	Aves,*Scytalopus*,*Eleoscytalopus*, Ramphastidae, toucans, phylogeny, phylogeography, cryptic diversification	S.L. Bonatto, T.R.O. Freitas, N.J.R. Fagundes
2016	Mammalia, placental, oxytocin and arginine vasopressin receptor evolution	M.C. Bortolini, F.M. Salzano
2004	Mammalia, Insectivora, origin	E. Eizirik
2002-present	Mammalia, Rodentia,*Ctenomys*,*Calomys*,*Zigodontomys*, extensive study involving many species, chromosome evolution, hybrids, phylogeny, phylogeography	T.R.O. Freitas
2002-present	Carnivora, Felidae, Mustelidae, Canidae, extensive study, many species, phylogeny, hybridization, phylogeography, implications for conservation.	E. Eizirik, T.R.O. Freitas
2015, 2017	Primates, oxytocin (OXT) and its receptor (OXTR), coevolution, paternal care in New World primates	M.C. Bortolini, F.M. Salzano

**Table 6:** Selected examples of human evolutionary studies performed by members of the Porto Alegre group (2002-present).

Year	Nature of the study	Contact
	1. **Non-Amerindian populations**	
2005, 2007, 2012, 2013	1.1. Extinct and extant, worldwide, comparison of *sapiens* with neandertals and denisovans, alternative models of human evolution, and variability in the low density lipoprotein receptor (*LDLR*) gene	N.J.R. Fagundes, S.L. Bonatto, M.C. Bortolini, F.M. Salzano
2003-2005, 2007, 2015	1.2. Latin America, studies in populations from Rio Grande do Sul, Porto Velho (Amazon), Uruguay, Argentina, Colombia, and Venezuela	M.C. Bortolini, F.M. Salzano, M.H. Hutz, S.M. Callegari-Jacques
2003-2005, 2007-2009, 2014	1.3. Interethnic admixture, Latin America, genetic and genomic approaches, many populations	M.C. Bortolini, F.M. Salzano, S.M. Callegari-Jacques
	2. **Amerindians**	
2002, 2006	2.1. General reviews	F.M. Salzano, S.M. Callegari-Jacques
2007, 2008, 2011, 2012, 2015	2.2. Prehistoric peopling of the Americas, general review, genetic and genomic approaches	M.C. Bortolini, F.M. Salzano, N.J.R. Fagundes, S.L. Bonatto
2002, 2008, 2010, 2012, 2013, 2015	2.3. Gene-culture coevolution, ecology, genetic/genomic relations with languages, other aspects of culture	M.C. Bortolini, F.M. Salzano, S.L. Bonatto, N.J.R. Fagundes
2006-2009, 2011	2.4. French Guiana, extensive investigation of its Amerindians, in close collaboration with colleagues from Toulouse, France	F.M. Salzano, S.M. Callegari-Jacques, M.H. Hutz, M.C. Bortolini, S.L. Bonatto
2002, 2007, 2008	2.5. Specific population approaches, extensive studies in Kaingang, Guarani, Xavante and Ache populations	F.M. Salzano, S.M. Callegari-Jacques, M.C. Bortolini, M.H. Hutz
2010, 2013, 2014, 2016	2.6. The immune system, Herpes Virus Type 8 prevalences, and different approaches on the variability of immune systems and its evolutionary significance	M.H. Hutz, F.M. Salzano, S.M. Callegari-Jacques
2002-2016	2.7. Specific systems approach	
	2.7.1. Autosome markers, more than 25 different systems	M.H. Hutz, S.M. Callegari-Jacques, M.C. Bortolini, S.L. Bonatto, F.M. Salzano
2004, 2012	2.7.2. Mitochondrial DNA	S.L. Bonatto, M.C. Bortolini
2002, 2003, 2008, 2009, 2011, 2012	2.7.3. Sex chromosomes, X and Y studies	M.C. Bortolini, M.H. Hutz, S.M. Callegari-Jacques, F.M. Salzano

Table 3 presents some selected examples of studies of general nature and of those involving microorganisms. Recently, Cazé *et al.* (2016) considered the question of factors affecting the genetic variability of the Atlantic Forest, with special reference to the refuge theory and river barriers.

Selected studies on plants are listed in Table 4. A large number of proteins of different types were surveyed in many species, searching for factors involved in their evolution. Specific studies in nine taxonomic families were indicated in the table, and specific mention can be made of a phylogenetic study with ecological niche modeling in the Myrtaceae (Turchetto-Zolet *et al.*, 2016), relating them with climate changes in the southern and northern Atlantic Forest.

Animal research, in species ranging all the way from Platyhelminthes to primates, is mentioned in Table 5. The Paired box (PAX) family of transcription regulators and developmental genes plays a key role in numerous stages of embryonic development, and its variability from Porifera to Vertebrates was investigated by Paixão-Côrtes *et al.* (2015). At another level, an analysis of the Drosophilid fauna was performed to address the question of the conservation units in the Caating biome (Oliveira *et al.*, 2016).

Selected examples of human evolutionary studies are given in Table 6. The whole genomes of *Homo sapiens*, *Homo neanderthalensis*, and Denisovans were searched by Paixão-Côrtes *et al.* (2013) to investigate the question of possible differences in cognitive ability between extinct and extant hominins. Results from 51 genes that affect this ability indicated similarity, with all the derived alleles being present in the three entities; while alternative models of human evolution were tested by Fagundes *et al.* (2007).

Latin American populations were extensively investigated during the period considered, and the question of interethnic admixture dynamics was addressed by Salzano and Sans (2014).

As for Amerindians, the prehistoric peopling of the Americas was examined in detail, using craniofacial morphology and uniparental genetic markers (González-José *et al.*, 2008). The French Guiana Amerindians were investigated using protein and DNA (autosome, mtDNA, Y-chromosomal) markers, and the fundamental question of the relationship between gene and culture was addressed in several publications, especially for the Xavante (Hünemeier *et al.*, 2012). These Amerindians were the subject of a whole book published by Coimbra Jr *et al.* (2002).

## The Porto Alegre Biological Evolution Workshops (PABEWs)

In 2006 our group, together with some other colleagues, considered it appropriate to start a cycle of international workshops in which key aspects of the evolutionary process could be discussed, where working hypotheses could be formulated and research projects delineated to answer questions. The invited persons, both Brazilian and foreigners, needed to have a wide vision of the evolutionary processes, which should be considered from a historical-philosophical point of view using empirical data from plant, animals, and humans. The first Workshop occurred in November, 2007, followed by four others in 2009, 2011, 2013, and 2015, always in November. Their format was also always the same: one-hour conferences, all by worldwide renown scholars, would be followed by 10 minutes of comments given by two specialists, followed by participation from the audience. The discussion period was always equal to that of the conference. The number of participants could not be more than 120, and attendance was subjected to previous selection, to assure that only persons already involved in evolutionary studies would attend.

The Fifth PABEW was held from November 9 to 11, 2015, in the Auditorium of our Department. It included 13 non-Brazilian speakers from Argentina, French Guiana, USA (3), UK (2), Norway, Switzerland (2), France, Spain, and Australia. Among the Brazilian commenting researchers 11 were from universities other than UFRGS. The meeting, as the previous ones, was a success for interchange of ideas and results, which in some instances led to the formation of joint research projects.

## Final message

As was emphasized at the beginning of this article, the process of formation of research groups is a slow one, and their maintenance is always in danger due to internal or external factors. These difficulties are especially notable in Third World countries, making the maintenance of our group for almost seven decades a remarkable event. It was, therefore, appropriate to review these accomplishments now, especially since Brazil is in a new period of economic difficulties, and there are generalized misunderstandings among our government officials about the importance of science. I finish this paper by expressing my hopes that this situation will be transitory, and that we and colleagues from other institutions will continue to contribute in a significant way to world knowledge.
